# Effect of a mobile mammography unit on participation and equity in breast cancer screening: a cluster randomised trial in Normandy, France

**DOI:** 10.1016/j.breast.2025.104686

**Published:** 2025-12-27

**Authors:** Gniré Koné, Séverine Beuriot, Ludivine Launay, Guy Launoy, Elodie Guillaume

**Affiliations:** a U1086 INSERM “ANTICIPE”, Caen Normandy University, Equipe Labellisée Ligue Contre le Cancer, Caen, France; b Centre de Lutte Contre le Cancer François Baclesse, Caen, France; c CHU de Caen Normandie, Caen, France

**Keywords:** Cluster randomised trial, Breast cancer screening, Mobile mammography, Social inequalities, Geographic inequalities

## Abstract

**Background:**

Participation in organised breast cancer screening (OBCS) in France has declined over the past decade. This study evaluated the contribution of mobile mammography units (MMUs) to increasing screening participation through a prospective cluster-randomised controlled trial conducted in France.

**Methods:**

This interventional study was conducted among the general population in four departments of the Normandy region. Areas located >15 min from a radiology centre were grouped into clusters and randomly assigned (1:1) to either an intervention or control arm. In total, 320 areas inhabited by 87,449 women aged 50–74 years were included. In the intervention arm, women whose last mammogram was performed at least 22 months earlier received, besides to the usual invitation, an appointment at the MMU sent by the regional screening management structure. The primary outcome was the BCS participation rate. A cluster-adjusted proportion test was used to compare participation between arms.

**Results:**

In the intervention arm, 22,964 women were screened out of the 38,382 invited, yielding a participation rate of 59.8 % vs 51.1 % in the control areas (25,099/49,067). The MMU intervention was associated with a statistically significant increase in participation of 8.7 % (p < 0.0001) compared with the control arm. In the intervention arm, women screened in the MMU tended to be younger and more deprived than those who opted for a radiology centre.

**Conclusions:**

The addition of an MMU to the OBCS programme in France significantly increased participation among women living furthest from radiology centres and can reduce social and geographic inequities.

## Highlights

• Socioterritorial inequalities exist in breast cancer screening participation.
• Mobile mammography units (MMUs) are used in various countries for screening.
• No RCTs have assessed the effectiveness of MMUs for breast cancer screening.
• Using MMUs can increase screening participation in deprived populations.
• Using MMUs with proportionate universalism can reduce socioterritorial inequalities.

## 1 Background

Breast cancer remains the most commonly diagnosed cancer and the leading cause of cancer-related mortality among women worldwide. To mitigate this burden, several countries have implemented organised breast cancer screening (OBCS) programmes aiming to reduce mortality through early detection. Achieving this requires robust public health strategies that ensure both high-quality health information for the target population and equitable access to screening services. However, most of these programmes operate on the principle of universality, ensuring equality but not necessarily equity.

Specifically in the European context, while many member states have implemented OBCS programmes, participation rates vary significantly. In France, in particular, OBCS was implemented nationwide since 2004, women aged 50–74 years at average risk are invited every two years to undergo a clinical breast examination and mammography at an accredited radiology centre. Screening participation rates differ widely; Nordic nations lead by example—notably Denmark (83.0 %), Finland (82.2 %), and Sweden (80.0 %)—closely followed by Malta (77.8 %) and Slovenia (77.2 %) [1]. However, France exhibits one of the lowest participation rates within the European Union (EU), standing at only 46 % in 2023, a figure substantially below the 70 % target set by the World Health Organisation and the EU [2].

This low rate highlights that non-attendance at breast cancer screening programmes remains a major public health challenge in many countries. Sociodemographic determinants of non-participation to the OBCS are well established. Studies consistently show that socioeconomic deprivation and geographic inequalities lead to lower screening uptake [[2], [3], [4], [5], [6], [7], [8]]. Inequalities are also spatial, associated with rurality and physical distance from health and radiology services [6,9]. Consequently, women who are socially disadvantaged and/or live in deprived or remote areas are less likely to participate in screening [[10], [11], [12], [13]].

Despite France's universal social protection system, several studies have demonstrated this association between social and spatial disparities and screening participation [10,12,13]. Notably, Rollet et al. [14] analysed 41 French departments, providing a detailed account of these socio-territorial inequalities. To address these inequalities, including the gradient in screening participation, a combination of universal and targeted public health programmes is essential. These should follow the principle of proportionate universalism, offering enhanced screening strategies to underserved populations to promote equity. Outreach services, such as mobile mammography units (MMUs), may be effective tools to increase participation in deprived areas identified through territorial approaches.

MMUs are used in several countries worldwide, including the US [15], Brazil [16], Canada (including Quebec), and at least seven European Union member states [17]. In some settings, such as Sweden, MMUs are routinely deployed to complement organised screening programmes [18], whereas in others, they are deployed only in specific regions [11,15,19]. To our knowledge, most of these studies have not used comparative designs and therefore do not meet the criteria for high-level evidence. Notably, only one BCS study was included in the 2016 Cochrane Review on mobile units [20].

In France, a 2017 retrospective study conducted in a rural department suggested that MMUs may help reduce social and geographic inequalities when deployed in underserved areas [11]. Most existing evaluations were not explicitly designed to address known inequalities or to set equity as a central objective. Consequently, high-quality evidence to inform public health decision-making remains limited. To our knowledge, no prospective controlled trial has assessed MMU's effectiveness in increasing participation or reducing social and geographic inequalities in BCS, either in France or elsewhere in Europe.

To evaluate the efficacy of this strategy in a French context, we conducted a cluster-randomized trial in remote areas of the Normandy Region between March 2022 and October 2023. The primary aim of this study is to quantify the effectiveness of the MMU intervention in improving breast cancer screening participation rates and, secondarily, to assess its impact on reducing social and territorial inequalities in screening access.

## 2 Methods

### 2.1 Study design

The Mammobile project was an interventional study conducted in the general population across four departments in the Normandy region (Eure, Calvados, Manche, and Seine-Maritime) between March 2022 and October 2023. The study used a cluster-randomised controlled trial design. Clusters were constructed by aggregating remote Ilot Regroupé pour l'information Statistique (IRIS) units located at least 15 min from an accredited radiology centre. These areas were randomised into two parallel arms in a 1:1 allocation ratio, comprising an intervention arm and a control arm. The initial protocol has been reported previously [21].

**Fig. 1:**
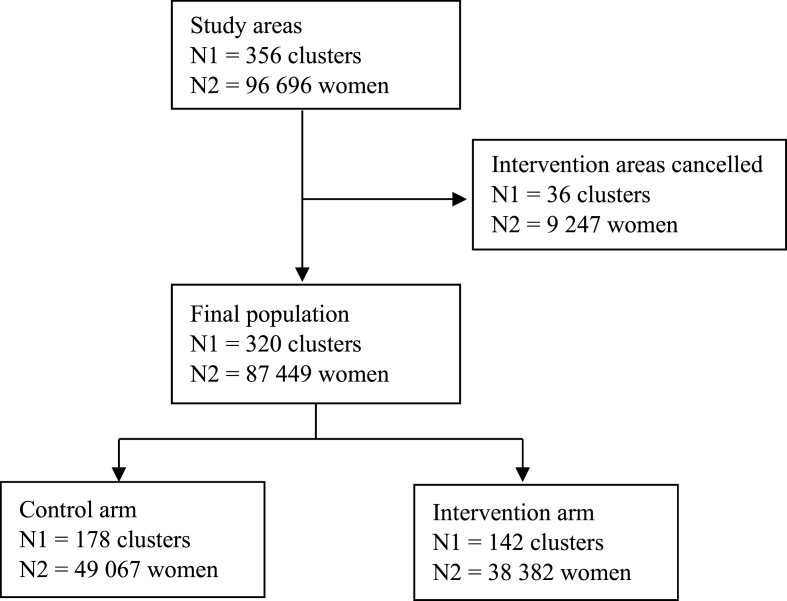
Flowchart of women's selection for inclusion in the study.

In total, 356 zones located at least 15 min from an accredited radiology centre were initially defined using a dedicated algorithm—178 in the intervention arm and 178 in the control arm—representing an estimated 45,275 and 46,707 eligible women, respectively. During implementation, several technical and logistical difficulties led to a reduction in the number of areas involved in the intervention (N = 142), resulting in a decreased number of eligible women (N = 38,382). The 36 intervention areas (Fig. 1) in which the MMU round was cancelled were excluded from statistical analyses. The control arm remained unchanged, comprising 178 areas with 49,067 eligible women.

**Fig. 2:**
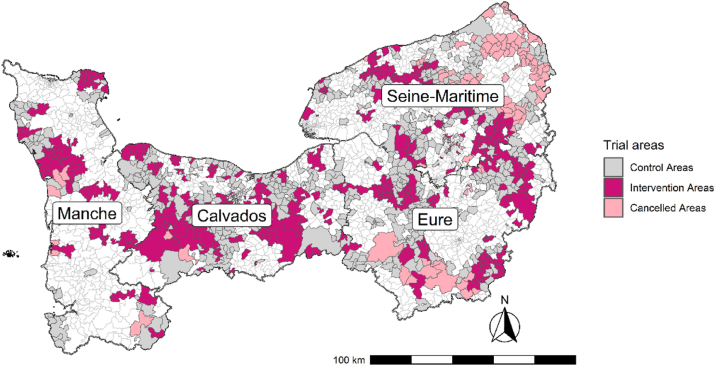
Geographical distribution of trial areas by allocation and inclusion status.

Fig. 2 shows the geographical distribution of included intervention areas (dark pink), excluded intervention areas (light pink), and control areas (grey). Table 1 presents the distribution of age and European Deprivation Index (EDI) quintiles across the excluded and included intervention clusters.

**Table 1:** Distribution of age and EDI quintiles in excluded and included clusters (intervention arm).

	Included clusters (*n* = 38,382)	Excluded clusters (*n* = 9239)	All (*N* = 47,621)
Age group (in years)			
50–54	8037 (20.9 %)	1863 (20.2 %)	9900 (20.8 %)
55–59	6347 (16.5 %)	1640 (17.7 %)	7987 (16.8 %)
60–64	6231 (16.2 %)	1590 (17.2 %)	7821 (16.4 %)
64–69	6191 (16.1 %)	1468 (15.9 %)	7659 (16.1 %)
70–74	11,576 (30.2 %)	2678 (28.9 %)	14,254 (29.9 %)
**EDI quintiles**			
Q1 (least deprived)	6897 (17.9 %)	425 (4.6 %)	7322 (15.4 %)
Q2	7514 (19.6 %)	1618 (17.5 %)	9132 (19.2 %)
Q3	7558 (19.7 %)	2399 (26.0 %)	9957 (20.9 %)
Q4	6855 (17.9 %)	2215 (24.0 %)	9070 (19.0 %)
Q5 (most deprived)	8593 (22.4 %)	2292 (24.8 %)	10,885 (22.9 %)
Missing	965 (2.5 %)	290 (3.1 %)	1255 (2.6 %)

EDI=European Deprivation Index.

### 2.2 Participants and procedures

Women eligible for OBCS were aged 50–74 years, with no personal history of breast cancer or specific cancer risk, and whose last mammography had been performed at least 22 months previously. In areas allocated to the intervention arm, women who had not undergone mammography since the usual invitation (n = 18,057) received an additional appointment at the MMU, issued by the screening management structure. This was provided alongside the standard invitation, and women retained the option of attending either the mobile unit or a radiology centre.

An MMU parking schedule was established for each department. The MMU was equipped with a mammography and an ultrasound scanner, and the healthcare team comprised a medical secretary, radiology technician, and radiologist. In one department (Eure), owing to a shortage of volunteer radiologists, screening in 33 areas was performed by a general practitioner, with mammogram readings deferred to a radiologist.

All mammograms were interpreted in accordance with national guidelines, including double reading of negative examinations. In intervention areas, MMU's presence was supported by communication materials and outreach activities to promote awareness and participation. The standard OBCS programme remained unchanged in areas assigned to the control arm.

### 2.3 Data sources and outcomes

Residential addresses were geocoded and georeferenced to calculate the road distance to the nearest radiology facility and to determine the deprivation level of each residence using the French version of the EDI (F-EDI). The principles and construction of this index, based on Eurostat and the 2011 French census data, have been described previously [21,22]. In this study, the index was divided into five quintiles, with quintile 1 representing the most privileged areas and quintile 5 the most disadvantaged.

As described in a previous publication [21], the primary objective was to assess the intervention's ability (a) to increase participation rates in remote populations and (b) to reduce socio-territorial inequalities in participation in OBCS across the Normandy region. The primary outcome was the screening participation rate, stratified by trial arm and cluster.

### 2.4 Statistical analysis

Descriptive data on trial areas and the number of eligible women in each arm are presented in Table 2. The distributions of age and F-EDI quintiles in the intervention and control arms are shown in Table 3.

**Table 2:** Distribution of areas and women in each department by study arm.

Department	Arm	Women (*n*)	*N* (%)	Areas (*n*)	*N* (%)
**Eure**	Control	14,783	27,460 (31.4)	58	103 (30.9)
	Intervention	12,677		45	
**Calvados**	Control	15,602	27,777 (31.7)	54	100 (30.0)
	Intervention	12,175		46	
**Manche**	Control	7542	13,516 (15.5)	30	57 (17.1)
	Intervention	5974		27	
**Seine-Maritime**	Control	11,140	18,696 (21.4)	43	73 (22.0)
	Intervention	7556		30	
**Total**		87,449		333^a^ (320)	

a 13 clusters covered two departments. Data are presented as n and N (%) unless otherwise stated.

**Table 3:** Distribution of age and EDI quintiles in the control and intervention arms.

	Control arm (*n* = 49,067)	Intervention arm (*n* = 38,382)	All (*N* = 87,449)
Age group (in years)			
50–54	11,274 (23.0 %)	9295 (24.2 %)	20,569 (23.5 %)
55–59	9431 (19.2 %)	7624 (19.9 %)	17,055 (19.5 %)
60–64	9761 (19.9 %)	7487 (19.5 %)	17,248 (19.7 %)
64–69	9602 (19.6 %)	7338 (19.1 %)	16,940 (19.4 %)
70–74	8999 (18.3 %)	6638 (17.3 %)	15,637 (17.9 %)
**EDI quintiles**			
Q1 (least deprived)	7718 (15.7 %)	6897 (17.9 %)	14,615 (16.7 %)
Q2	9776 (20.0 %)	7514 (19.%)	17,290 (19.8 %)
Q3	8782 (17.9 %)	7558 (19.7 %)	16,340 (18.7 %)
Q4	12,730 (25.9 %)	6855 (17.9 %)	19,585 (22.4 %)
Q5 (most deprived)	8385 (17.1 %)	8593 (22.4 %)	16,978 (19.4 %)
Missing	1676 (3.4 %)	965 (2.5 %)	2641 (3.0 %)

Data are *n* (%). EDI=European Deprivation Index.

In the intervention arm, women who had not attended screening following their usual invitation and were subsequently invited to the MMU represented 47.6 % of the arm's eligible population (46.7 %, 46.4 %, 54.4 %, and 42.9 % in Eure, Calvados, Manche, and Seine-Maritime, respectively). Participation rates were compared between the two arms using the entire eligible population. A flowchart of the selection process is provided in Fig. 1.

To ensure comparability between study arms, we defined matched participation periods in the control arm corresponding to the MMU implementation period in each department.

**Fig. 3:**
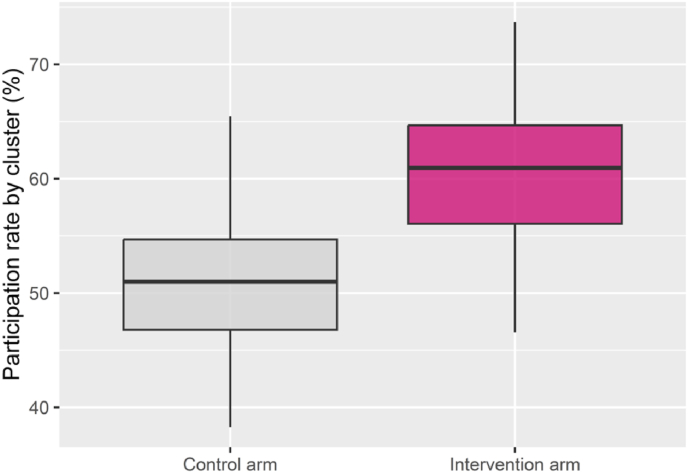
Participation rate by area in each arm.

Participation rates were calculated as the number of mammographies performed during the participation period divided by the number of eligible women during the invitation period, per department. Descriptive analyses of participation rates are shown in Table 4. Area-level participation rates are presented in Fig. 3, with summary data by arm in Table 4. The screening location for women who received a supplementary MMU invitation in intervention arm is shown in Table 5.

**Table 4:** Participation rates in the control and intervention arms by department.

Department	Control arm (*N* = 49,067)	Intervention arm (*N* = 38,382)	Absolute difference in participation	
			Percentage	P-value
**Eure**	7472 (50.5 %)	7361 (58.1 %)	7.5 %	<0.0001
**Calvados**	7918 (50.7 %)	7127 (58.5 %)	7.8 %	<0.0001
**Manche**	3618 (47.9 %)	3666 (61.4 %)	13.5 %	<0.0001
**Seine-Maritime**	6091 (54.7 %)	4810 (63.6 %)	8.9 %	<0.0001
**All**	25,099 (51.1 %)	22,964 (59.8 %)	8.7 %	<0.0001

Data are presented as n (%), where n is the number of women participating in the screening. Participation rates were compared using a cluster-adjusted proportion test (intraclass correlation: 0.0118 for intervention, 0.0139 for control).

**Table 5:** Screening location for women who received a supplementary MMU invitation (intervention arm only).

Department	Supplementary Invitation (n)	Screening site	
		RC	MMU
**Eure**	5921	760 (38.8 %)	1196 (61.2 %)
**Calvados**	5644	904 (42.4 %)	1228 (57.6 %)
**Manche**	3249	406 (29.8 %)	954 (70.2 %)
**Seine-Maritime**	3243	485 (39.5 %)	743 (60.5 %)
**All**	18,057	2555 (38.3 %)	4121 (61.7 %)

Data are presented as n (%). RC=Radiology Centre. MMU=Mobile Mammography Unit.

A cluster-adjusted proportion test was used to compare participation rates between arms. The intraclass correlation coefficients were 0.0118 and 0.0139 in the intervention and control arms, respectively. Among women who received an additional MMU invitation, a chi-squared test of independence was used to compare age and level of social deprivation by screening location (MMU vs radiology centre), as shown in Table 6.

**Table 6:** Distribution of age and EDI quintile by screening site (women with supplementary invitation only).

	No screening (n = 11,381)	RC (n = 2555)	MMU (n = 4121)	All (N = 18,057)	P-value
**Age group (in years)**					<0.0001
50–54	2945 (25.9 %)	607 (23.8 %)	1089 (26.4 %)	4641 (25.7 %)	
55–59	2498 (21.9 %)	512 (20.0 %)	896 (21.7 %)	3906 (21.6 %)	
60–64	1988 (17.5 %)	548 (21.5 %)	828 (20.1 %)	3364 (18.6 %)	
64–69	2108 (18.5 %)	486 (19.0 %)	768 (18.6 %)	3362 (18.6 %)	
70–74	1842 (16.2 %)	402 (15.7 %)	540 (13.1 %)	2784 (15.4 %)	
**EDI quintiles**					<0.0001
Q1 (least deprived)	1837 (16.1 %)	513 (20.1 %)	672 (16.3 %)	3022 (16.7 %)	
Q2	2317 (20.5 %)	512 (20.0 %)	819 (19.9 %)	3648 (20.2 %)	
Q3	2306 (20.2 %)	501 (19.6 %)	845 (20.5 %)	3652 (20.2 %)	
Q4	2192 (19.3 %)	432 (16.9 %)	881 (21.4 %)	3505 (19.4 %)	
Q5 (most deprived)	2727 (23.9 %)	597 (23.4 %)	904 (21.9 %)	4228 (23.4 %)	

Data are presented as n (%). Chi-squared test of independence. RC=Radiology Centre. MMU=Mobile Mammography Unit. EDI=European Deprivation Index.

## 3 Results

The number of areas and eligible women included in each arm across the four departments is reported in Table 2, and the women's selection flowchart is shown in Fig. 2. The analysis covered 320 remote areas—142 in the intervention arm and 178 in the control arm—including 103, 100, 57, and 73 areas in Eure, Calvados, Manche, and Seine-Maritime, respectively. These areas corresponded to populations of 27,460 women in Eure, 27,777 in Calvados, 13,516 in Manche, and 18,696 in Seine-Maritime.

The demographic characteristics of the 87,449 women included in the analysis were generally balanced between study arms (Table 3). There was no statistically significant difference in age distribution between the control and intervention arms. Overall, 20,569 women (23.5 %) were aged 50–54 years, and 15,637 (17.9 %) were aged 70–74 years. However, the distribution of social deprivation levels differed slightly, despite randomisation. The proportion of women residing in the most deprived quintile (Q5) was higher in the intervention arm (8593 women, 22.4 %) than in the control arm (8385 women, 17.1 %). In contrast, 7718 women (15.7 %) in the control arm lived in the most privileged quintile, compared to 6897 women (17.9 %) in the intervention arm. Overall, 14,615 women (16.7 %) lived in the most privileged quintile, and 16,978 (19.4 %) in the most disadvantaged.

Table 4 presents the participation rates in the OBCS in the control and intervention arms during the defined periods for each department. Overall, of the 49,067 women invited to OBCS in the control areas across the four departments, 25,099 underwent screening mammography, resulting in a participation rate of 51.1 % for the campaign periods included in the analysis. Manche recorded the lowest participation rate (47.9 %), whereas Seine-Maritime had the highest (54.7 %).

In the intervention arm, 22,964 women were screened out of the 38,382 invited, yielding a participation rate of 59.8 %. Among the 18,057 women who received an additional invitation to attend the MMU, 6676 (37 %) underwent screening. Of these, 4121 (61.7 %) were screened in the MMU and 2555 (38.3 %) at a radiology centre (Table 5). The lowest participation rate in the intervention arm was recorded in Eure (58.1 %), and the highest in Seine-Maritime (63.6 %). In total, 160 women in the control areas underwent mammography via the MMU despite not being scheduled for MMU screening. These women were not refused screening for ethical reasons and were retained in the control group for analysis.

The MMU intervention was associated with a statistically significant increase in participation of 8.7 % (p < 0.0001) compared with the control arm. This increase was achieved by targeting only 47.6 % (18,057/38,382) of eligible women—specifically, those who had not attended following the standard invitation. Across departments, the increase in participation ranged from 7.5 % to 13.5 %, with the highest difference observed in Manche (13.5 %). All departmental increases were statistically significant (p < 0.0001).

Fig. 3 presents the distribution of participation rates by cluster in both study arms. In the control arm, 75 % of clusters had participation rates below 55 %. By contrast, in the intervention arm, 50 % of clusters achieved rates above 61 %. The lowest participation rate per cluster was 47 % in the intervention arm and 38 % in the control arm. In some intervention clusters, participation reached 78 %, whereas in the highest-performing control clusters, the rate was 66 %.

Table 6 presents the age and EDI distribution of women who received a supplementary invitation, stratified by screening location. Among women screened in the MMU, 48 % were aged under 60, compared with 43 % of those who opted for a radiology centre. Among radiology centre attendees, 15 % were aged 70–74, whereas in the MMU group, this figure was 13 %. Regarding deprivation, 43 % of women screened in the MMU lived in the most deprived areas. Conversely, 40 % of radiology centre users lived in the most privileged areas, compared with 36 % of MMU users.

## 4 Discussion

This study demonstrates that the integration of an MMU into existing OBCS programme in France can reduce territorial inequalities in participation, increasing uptake by an average of 8.7 % in remote areas furthest from radiology centres.

To our knowledge, this is the first prospective cluster randomised controlled trial in Europe to evaluate the effectiveness of an MMU in enhancing screening participation. The trial design allows attribution of the observed directly to the intervention, providing high-level evidence. Conversely, the women who contributed to the 8.7 % increase are those who would likely not have been screened without the intervention. Of this 8.7 % gain, 5.4 % can be attributed to the use of the MMU itself, representing the proportion of participants who attended the MMU (61.7 %; see Table 5). The remaining 3.3 % reflects women who preferred to attend a radiology centre, suggesting that additional invitation, rather than MMU offering, influenced their decision.

These findings also indicate that the MMU intervention reduced the social gradient in screening participation. Women in the most disadvantaged quintiles were more likely to respond to the MMU invitation. Among those who had the option to choose between the MMU and a radiology centre, socially disadvantaged women were more likely to attend the MMU.

Several factors potentially threatened group comparability. First, although randomisation ensured comparability between arms regarding age and distance to the nearest radiology centre, the intervention arm included a greater proportion of deprived areas. This imbalance introduces a conservative bias, as higher deprivation usually means lower uptake. Second, technical and logistical issues cancelled the intervention in 36 areas. These excluded areas were more deprived, but age comparability was not affected. Third, 160 control arm women were screened by the MMU. Retaining them introduces contamination, also representing a conservative bias. Fourth, although this was a randomised trial, it was implemented under real-world conditions in the general population. The intervention varied slightly across departments depending on contextual factors. For instance, departmental administrative support for community engagement and communication differed, as did the composition of mammography teams. Table 4 presents detailed department-level results, showing that the intervention arm consistently achieved more than a 5 % increase in participation, reaching up to 13.5 % in one department. In some zones, participation in the intervention arm exceeded the 70 % threshold recommended by the European Commission for effectively reducing breast cancer mortality (Fig. 3). Notably, overall screening uptake in both arms exceeded 50 %, consistent with National Public Health Agency reports for 2022–2023, which indicate that participation in the Normandy region was higher than the national average. Radiologist involvement also varied across departments. In Eure, the absence of radiologists was mitigated by the participation of general practitioners (GPs), who conducted the screening procedure in 33 areas. This substitution led to delays in mammogram interpretation. Further analyses will explore differences in performance and screening quality between radiologists and GPs.

Beyond the previously mentioned limitations, several additional factors regarding the external validity and design of our trial warrant emphasis. The generalisability of our findings should be interpreted with caution due to the intrinsically rural context of the Normandy region, which contrasts with other French territories. The impact of the intervention could differ significantly in more urban or semi-urban territorial settings where geographical access to conventional radiology centres is less restrictive. The scale of the experiment was constrained, targeting only approximately 17 % of the overall population eligible for the OBCS in the four concerned departments. This limited scope may have understated the intervention's true potential. It is plausible that a permanent and larger-scale integration of the device into the usual OBCS organisation would yield a more substantial and sustained gain in participation.

The strategy for targeting women for screening within the MMU constitutes a key methodological limitation. Our protocol offered MMU appointments only to women in the Intervention areas who had not undergone a mammogram in the previous 22 months. Consequently, women residing in these areas with up-to-date screening were systematically excluded from participating in the intervention. A more systematic and comprehensive targeting approach would maximise both the unit's operational throughput and the volume of mammograms performed. This strategy would entail offering all women eligible for OBCS in remote areas the choice of receiving their mammogram at either the MMU or the radiology centre. This would thereby reinforce the overall impact on population screening coverage.

Finally, we were unable to assess the quality of the screening performed within the MMU compared to that of accredited radiology centres. This lack of data represents a significant clinical limitation, as it prevents a comprehensive assessment of the device's benefit-risk ratio. However, we confirm that this specific quality assessment is a critical next step and is planned for completion once the requisite data becomes available. Furthermore, although socio-economic deprivation is a recognised determinant of non-participation in screening, our protocol focused exclusively on geographical remoteness as the criterion for defining the trial areas. The potential impact of a combined targeting strategy focusing on areas suffering from both geographical isolation and deprivation could not be evaluated in this study. This represents an essential avenue for future research concerning health equity.

MMUs are used in many countries, either as a primary modality or as an adjunct to OBCS. Several studies have demonstrated their utility in reaching populations underserved due to geographic remoteness or socioeconomic disadvantage. The majority of this evidence comes from the United States, where, despite the recommendations of the US Preventive Services Task Force, no universal national OBCS programme exists. In the US context, underserved populations are often defined by ethnicity or inclusion in public health insurance schemes such as Medicare.

A 2018 review by Vang et al. [23] focused on American studies and reported that MMU programmes consistently reached underserved women. More recent studies have shown that MMUs improved access to screening in underserved areas of Texas [24] and that women utilising these services often had specific socioeconomic characteristics that would have otherwise placed them at risk of non-participation [25]. Ozcan et al. [26] also proposed MMUs as a strategy to reduce screening disparities in the US. Similar initiatives have been implemented in Brazil [18] and Taiwan [27].

In Europe, however, MMU-based studies are scarce. Most European countries offer population-wide, high-quality screening via radiologists and public hospitals as part of universal healthcare systems. Nonetheless, territorial and social inequalities persist despite the existence of these systems. In this context, the question is not whether screening is available, but rather what additional value an MMU can offer—specifically, whether it increases participation and reduces inequality.

While MMUs have been incorporated into screening strategies in several European regions, no prospective trials have been conducted to quantify their impact. To our knowledge, our study is the first to measure the additional participation attributable to the MMU—beyond the standard screening offer—using a randomised controlled trial framework and applying the principle of proportionate universalism in a targeted population.

The trial was conducted under real-world conditions, embedded within standard screening practices. It demonstrated the MMU’s capacity to increase participation and reduce socioeconomic disparities in access to screening in remote areas. The broader public health impact of implementing MMUs in France will depend largely on the incremental cost of reaching additional women. Forthcoming results from the cost-effectiveness analysis will be crucial in informing the potential scale-up of MMU use.

In a previously published retrospective analysis of a similar intervention in France, the mean incremental cost per additional screening was €610.69 [28]. This cost was lower among women living >15 km from a radiology centre (€289.57) and those residing in deprived areas (€347.96). Although cost-effectiveness and comparisons across countries are challenging because of variations in healthcare systems and pricing structures, these estimates are comparable to findings from an American study by Naeim et al. [29] ($264), and to other reminder-based interventions, such as telephone or digital follow-up [30].

A key issue affecting the scalability of MMU deployment is staffing. Due to the experimental, time-limited nature of the trial, temporary staff—including radiologists, radiology technicians, and non-medical personnel—had to be recruited and trained for each department. To integrate the MMUs sustainably into the national screening infrastructure, an operational model involving permanent medical and non-medical personnel will be necessary. This could reduce operating costs, enhance service continuity, and improve quality assurance.

## 5 Conclusion

This prospective, cluster-randomised controlled trial—the first of its kind in Europe—provides strong evidence supporting the integration of an MMU into France's organised breast cancer screening programme.

Our robust findings confirm that the MMU is an effective and equitable strategy for addressing persistent screening inequalities within a universal healthcare system. The intervention offered significant added value beyond standard screening by increasing participation and reducing socioeconomic disparities in access, particularly among remote populations. These results validate the principle that proximity-based care delivery is crucial for reaching underserved groups. By prioritising those with the greatest need, the MMU model operationalises the principle of proportionate universalism to mitigate breast cancer screening inequalities.

This study supports the wider implementation of the MMU model—not as a replacement for, but as a complementary component of—established OBCS programmes. The demonstrated ability to reach underserved populations underscores the MMU's role as a key instrument for promoting health equity. Ensuring the availability of qualified professionals to staff MMUs is essential to maintain service quality, ensure continuity of care, and maximise the public health impact of this equity-focused intervention. Further research, particularly cost-effectiveness analyses, will be crucial to guide national scale-up decisions.

## CRediT authorship contribution statement

**Gniré Koné:** Writing – review & editing, Writing – original draft, Software, Methodology, Formal analysis, Data curation. **Séverine Beuriot:** Project administration. **Ludivine Launay:** Formal analysis. **Guy Launoy:** Writing – review & editing, Visualization, Supervision, Methodology, Investigation, Conceptualization. **Elodie Guillaume:** Writing – review & editing, Visualization, Supervision, Methodology, Investigation, Conceptualization.

## Ethics approval and consent to participate

The study was approved by the National Commission of Informatics and Liberties agreement (CNIL; approval no. 921203) and registered on ClinicalTrials.gov (NCT05164874). We obtained positive agreement from the ethics committee CEREES (1477006 Ter).

## Funding sources

The project is funded by the French National Cancer Institute, by the regional health agency in charge of the OBCS application and by the association Ruban Rose (https://www.cancerdusein.org/). U1086 ANTICIPE is a League Against Cancer Labelled Team. The funders had no role in the design of the study, data collection, data analysis, data interpretation, or writing of the manuscript.

## Declaration of competing interest

The authors declare that they have no known competing financial interests or personal relationships that could have appeared to influence the work reported in this paper.

## Data Availability

Certain details of the datasets used in this study are available upon request from the screening management structure. All protocol data can be obtained upon request.
